# Draft Genome Sequence of *Lysinibacillus xylanilyticus* SR-86

**DOI:** 10.1128/genomeA.01317-16

**Published:** 2016-11-23

**Authors:** Michael E. Loscar, Christopher Huptas, Mareike Wenning, Volker Sieber, Jochen Schmid

**Affiliations:** a Chair of Chemistry of Biogenic Resources, Technical University of Munich, Straubing, Germany; b Chair for Microbial Ecology, Technical University of Munich, Freising, Munich, Germany

## Abstract

*Lysinibacillus xylanilyticus* belongs to the family *Bacillaceae* and was first described in 2010 with the type strain *L. xylanilyticus* XDB9. It is able to both degrade xylan and use it as the sole carbon source. Here, we describe the 4.8-Mb genome of the strain *L. xylanilyticus* SR-86, which was isolated from organic waste.

## GENOME ANNOUNCEMENT

*Lysinibacillus xylanilyticus* SR-86 was obtained by classical microbiological isolation techniques from organic waste. The strain was identified as *L. xylanilyticus* by a 1,498-bp PCR fragment of the 16S rRNA, which was analyzed using the EzTaxon server (1) with 100% pairwise similarity of the reference type strain (2). The genomic DNA was isolated using the DNeasy blood and tissue kit (Qiagen, USA) of a culture grown overnight according to the manufacturer’s protocol for Gram-positive microorganisms (3). PCR-free library preparation (IS1) was performed with an optimized protocol as described by Huptas et al. (4). Sequencing was carried out on the Illumina MiSeq system using V3 chemistry.

Read data were trimmed (10 nucleotides [nt] from the 5′ end and one nt from the 3′ end) and quality filtered (options: -l 80.0, -s 20) using the NGS QC Toolkit version 2.2.3 (5). High-quality read pairs (2 × 200 nt) were visually inspected using FastQC version 0.11.4 (6) prior to assembly with SPAdes version 2.5.1 (7) applying the *k*-mer combination <21, 33, 55, 77, 99, 127>. The draft genome assembly contained 49 contigs with an *N*_50_ of 412,414 nt. With 1,361,289 high-quality read pairs, the achieved sequencing depth was ~114-fold considering an assembly size of 4,763,528 nt.

Annotation was performed by uploading the assembly to the RAST server (8), which detected 4,791 coding sequences in 462 subsystems and 102 RNA genes. A total number of 168 genes for RNA metabolism were detected by RAST analysis (9).

Recently, a genome from *L. xylanilyticus* DSMZ 23493 was published (10). We compared the annotated sequences of strain DSMZ 23493 with that of SR-86 using the RAST server. In total, 5,312 entries were compared, of which 1,167 in SR-86 did not have a corresponding entry in DSMZ 23493. The GC content is similar, with 36% for SR-86 and 36.7% of DSMZ 23493. The genome size of SR-86 is smaller, with approximately 4.8 Mb compared to 5.2 Mb.

### Accession number(s).

This whole-genome shotgun project has been deposited at DDBJ/ENA/GenBank under the accession number MDDN00000000. The version described in this paper is the first version, MDDN01000000.
